# Interdisciplinary surgical approach enables complete tumor resection with preservation of neurological function in specific conditions of pediatric solid malignancies

**DOI:** 10.1007/s00432-022-04273-x

**Published:** 2022-09-21

**Authors:** Cristian Urla, Jörg Fuchs, Alexander Grimm, Andreas Schmidt, Jürgen Schäfer, Martin U. Schuhmann, Steven W. Warmann

**Affiliations:** 1 Department of Pediatric Surgery and Pediatric Urology, University Children’s Hospital of Tuebingen, Hoppe-Seyler-Strasse 3, 72076 Tuebingen, Germany; 2 Neuromuscular Division, Department of Neurology, University Hospital of Tuebingen, Tuebingen, Germany; 3 Department of Diagnostic and Interventional Radiology, University Hospital of Tuebingen, Tuebingen, Germany; 4 Division of Pediatric Neurosurgery, Department of Neurosurgery, University Hospital of Tuebingen, Tuebingen, Germany; 5 Center of Neurofibromatosis, Center of Rare Diseases, University Hospital of Tuebingen, Tuebingen, Germany

**Keywords:** Pediatric, Solid tumors, Interdisciplinary surgical approach, Preservation of neurological function

## Abstract

**Purpose:**

Success of pediatric solid tumor surgery is regularly hampered by infiltration of essential neurovascular structures. A surgical dilemma arises when imaging data suggest a conflict between complete resection and preservation of neurological function. The aim of the study was to analyze data of children harboring tumors with involvement of neurovascular structure treated by an interdisciplinary pediatric surgical/neurosurgical team.

**Methods:**

We retrospectively analyzed data of 25 children undergoing surgery for solid tumors, in whom preoperative imaging showed a relevant involvement of nerve structures. Surgery was simultaneously performed by a pediatric onco-surgeon and a pediatric neurosurgeon with peripheral nerve expertise, including intraoperative electrophysiological monitoring.

**Results:**

The following tumors were treated: NF1 associated neurofibromas (10), neuroblastomas (5), desmoid tumors (2), MPNST (2), ganglioneuroma (1), Ewing sarcoma (1), infantile fibromatosis (1), PNET (1), rhabdomyosarcoma (1), angiolipoma (1). The most frequent tumor localizations were the pelvis (*n* = 7) and retroperitoneal region (*n* = 6). Median age at surgery was 8 years (1.5–16). Macroscopically complete tumor resection was achieved in 24/25 patients. In 2/4 patients with limb tumors an amputation was planned externally. In both, a limb-salvage procedure was possible. Transient postoperative neurological deficits occurred in 2/25 patients. Four patients had tumor relapses. All but one are alive after a median follow-up of 46 months (2–155).

**Conclusions:**

Simultaneous interdisciplinary pediatric surgical/neurosurgical approach enables radical tumor resection with preservation of neurological function in patients suffering from solid tumors with involvement of relevant neurovascular structures. This approach should be performed by experienced surgeons in reference pediatric onco-surgical centers.

## Introduction

The outcome of children with solid tumors has improved significantly over recent years as a result of several national and international cooperative group treatment protocols, and due to the progress made in all of the disciplines involved in the treatment (Fuchs et al. 2012; Schmidt et al. 2018).

Complete resection of primary tumors and metastases represents the primary goal of the surgical procedures and is usually crucial for the prognosis of the patients. A correctly and consistently applied surgical strategy can help to reduce the amount of adjuvant therapies (chemotherapy, radiotherapy), and mitigate their consequences (Schmidt et al. 2018).

The success of tumor surgery in children is regularly hampered by affection of essential organs and neurovascular structures. Imaging data often suggest a conflict between complete tumor resection and preservation of nerve function or organ perfusion, if there is an immediate involvement of peripheral nerves and/or vascular structures. This generates a conflict between the necessity of a radical resection and the avoidance of mutilating procedures, thus providing a high-quality of life for the patients.

Simultaneous interdisciplinary surgery combining principles of radical pediatric onco-surgical and neurosurgical techniques for nerve preservation or nerve reconstruction, possibly contributes to achieving local control of tumors with involvement of neuronal structures and provide preservation or restoration of nerve function. This approach is associated with a higher logistic effort and requires a specific infrastructure. There are no data in the literature regarding the usefulness of an interdisciplinary surgical approach in affected children. Therefore, the aim of the present study was to analyze the data of children suffering from solid tumors with involvement of nerve structures treated by an interdisciplinary team of pediatric surgeons and pediatric neurosurgeons in a reference pediatric onco-surgical center in Germany.

## Patients and methods

### Patients

We evaluated data of patients with solid tumors undergoing surgery by a team including two senior pediatric surgeons (JF and SWW) and a senior pediatric neurosurgeon with additional expertise in peripheral nerve surgery (MUS.). In patients with solid tumors, other than NF1, the resection of the tumor was performed by the pediatric surgeon, while the neurosurgeon dissected and/or resected the encased nerve structures and performed the reconstruction of the involved nerves. In patients with NF1, the resection was performed either by the neurosurgeon (the pediatric surgeon only exposing the anatomical region) or combined, if a thoracoscopic approach was feasible.

A retrospective review of patient’s records was carried out. The study was approved by the local ethical committee (number 865/2021BO2). The study period was October 2008–May 2020. The following inclusion criteria were applied: age at surgery ≤ 18 years, diagnosis of extracranial pediatric solid tumor with involvement of relevant nerve structures, and tumor resection simultaneously performed by a pediatric surgical/neurosurgical team.

The indications for combined surgery were: surgical resection indicated according to the corresponding protocol, relevant involvement (with possible infiltration) of functionally important nerves on preoperative imaging, radical resection necessary from an oncological point of view despite involvement of neuronal structures. In patients suffering from neurofibromatosis type I (NF1) the indication for surgery was based on increased metabolism in PET/CT (Positron Emission Tomography/Computed Tomography) or PET/MRI (Positron Emission Tomography/Magnetic Resonance Imaging) or rapid increase in size (suggesting malignant transformation), or clinical symptoms from nerve affection.

In all patients suffering from malignant solid tumors, the (neo)adjuvant treatment was given according to the respective protocol of German Society of Pediatric Oncology and Hematology (GPOH).

Data were analyzed with special regard to patients’ and tumor characteristics, encased nerve structures, surgical procedures, surgical radicality, intra- and postoperative complications, as well as functional and oncological outcome. Data regarding race/ethnicity was not available.

### Radiologic workup

Preoperatively, the extent of the tumors was assessed using high-field MRI, computed tomography, PET/MRI or PET/CT. Ultrasound of peripheral nerves in the tumor region was additionally performed the day before surgery (AG) together with the neurosurgeon (MUS), if the nerve was accessible for ultrasound, to clarify the extent of nerve involvement and the exact loco-regional relation between nerve and tumor.

Postoperative complications were classified according to the system proposed by Dindo and Clavien (Dindo et al. 2004; Clavien et al. 1992).

### Statistical analysis

Follow-up time was regarded as period from surgery onwards. Demographic data are reported as medians (interquartile ranges). Statistical analyses were performed using SPSS software (version 26.0, IBM Corp. Armonk, New York, USA).

## Results

### General aspects

Simultaneous pediatric surgical/neurosurgical operations were performed in 25 children (18 female, 7 male). Patient’s data are presented in Table 1. Median age at operation was 8 years (range 1.5–16 years). Median duration of surgery was 190 min (58–548). Median hospital stay was 8 days (4–27), median stay on intensive care unit was 1 day (0–4 days). The most frequent tumor localization was the pelvis (*n* = 7), followed by the retroperitoneal region (*n* = 6), and the thorax (*n* = 5) as shown in Table 1. Median follow-up was 46 months (2–155).

**Table 1:** Patient’s characteristics

ID	Diagnosis	Age at surgery (years)	Operation time (minutes)	ICU stay (days)	Hospital stay (days)	Follow-up (months)	Localization
1	AL	3	308	0	11	13	Extremities
2	DT	12	330	0	8	139	Extremities
3	DT	8	548	3	20	105	Pelvis
4	ES	2	227	4	10	32	Thorax
5	GN	7	318	4	27	28	Pelvis
6	IF	2	285	1	7	51	Cervical
7	MPNST	16	180	0	15	138	Pelvis, extremities
8	MPNST	13	211	1	9	155	Retroperitoneal, extremities
9	NB	2	295	1	14	21	Thoracoabdominal
10	NB	1,5	210	0	8	92	Retroperitoneal
11	NB	3	293	2	13	56	Pelvis
12	NB	2	205	0	8	40	Pelvis
13	NB	1,5	190	1	10	10	Retroperitoneal
14	PNF	10	145	1	7	33	Thorax
15	PNF	10	183	0	10	108	Thorax
16	PNF	16	62	1	10	46	Thorax
17	PNF	14	58	0	6	87	Pelvis, extremties, inguinal
18	PNF	12	272	1	6	45	Pelvis
19	PNF	13	93	0	4	77	Retroperitoneal, thorax
20	PNF	15	79	1	10	52	Thorax
21	PNF	8	129	1	6	6	Abdominal wall
22	PNF	15	150	1	6	4	Retroperitoneal
23	PNF	11	140	1	7	2	Retroperitoneal
24	PNET	3	188	0	8	123	Extremities
25	RMA	4	89	0	4	12	Extremities

*AL* angiolipoma, *DT* desmoid tumor, *ES* Ewing sarcoma, *GN* ganglioneuroma, *IF* infantile fibromatosis, *MPNST* malignant peripheral nerve sheath tumor, *NB* neuroblastoma, *PNF* neurofibromatosis type I associated plexiforme neurofibroma, *PNET* peripheral neuroectodermal tumor, *RMA* alveolar rhabdomyosarcoma

### Surgical aspects

**Fig. 1:**
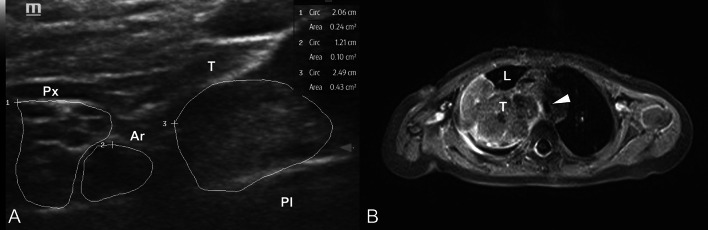
(**A**) Short-axis from the supraclavicular fossa including all cords of the brachial plexus (Px) with its honey comb aspect, next to the subclavian artery (Ar). The tumor mass (T) (hyperechoic, no fascicular structure in it) is well distinguishable by ultrasound, however owing the high resolution the real distance of the tumor and nerve is below 5 mm; (**B**) Axial view of the MRI imaging of the same patient showing the intrathoracic extension of the tumor (T) with infiltration of the trachea (white arrow)

Preoperatively, the patients with tumors located in the cervical region or at the extremities underwent a detailed high-resolution nerve ultrasound evaluation performed by an expert neuro-sonographer (AG) (Fig. 1). The nerve ultrasound was carried out in the presence of the interdisciplinary surgical team to assess the possible intraoperative risk profile, plan the appropriate surgical strategy (e.g. route of access, sural nerve excision/donation for graft repair), and for adequate family counseling.

**Fig. 2:**
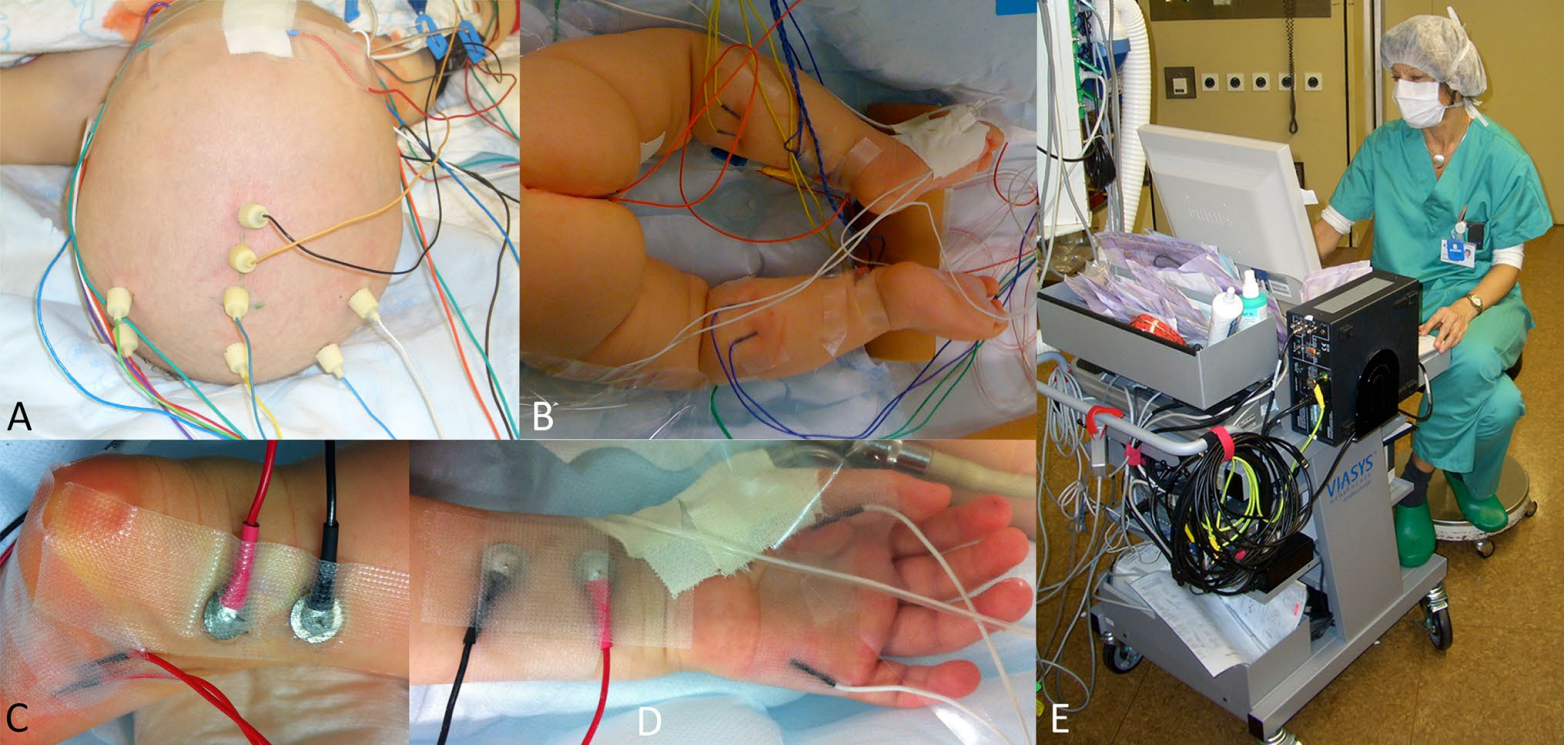
Set up for intraoperative evoked potential neuromonitoring by SEP and MEPs in case of involvement of brachial or lumbo-sacral plexus. (**A**) scalp screw electrodes for MEP stimulation and SEP recording; (**B**) peripheral muscle electrode positioning for MEP recording at the lower extremities. Visible Electrodes are placed in quadriceps muscle (L4), anterior tibial muscle (L5), gastrognemius muscle (S1), and anal sphincter (S3/S4); (**C**) Positioning of nerve stimulators (black, red) for posterior tibial nerve SEP generation and muscle electrodes to abductor hallucis muscle for MEP recording (S2); (**D**) Position of median nerve stimulators (black, red) for upper extremity SEP generation and muscle electrodes to adductor policis and abductor digit minimi muslces for MEP recording of lower brachial plexus (C8 root and inferior trunk); (**E**) Neurophysiologist interpreting the intra-operative monitoring results during surgery

Intraoperatively, a peripheral nerve stimulator for direct motor response monitoring was continuously available in all patients. Additionally, in 14/25 cases, intraoperative SEP/MEP monitoring (sensory and motor evoked potentials) was used (Fig. 2). SEP of median or tibial nerve were used when the brachial or lumbo-sacral nerve plexus (or its contributing roots) were involved. The muscles incorporated into MEP monitoring were determined by the involved region. In case of involvement of lumbo-sacral plexus, to monitor bladder function, the anal MEPs and the bulbus-cavernosus reflex (BCR) were included.

In six patients with NF I the pediatric surgeon exposed the tumor region, while the neurosurgeon performed the intracapsular tumor removal. In the remaining cases, the tumor resection was carried out by the pediatric surgeon, while the neurosurgeon dissected the relevant neural structures from the tumor, or resected the tumor components attached to the nerves (in some case including the perineurium). In one case, an en-bloc resection with immediate nerve reconstruction by sural nerve grafting was performed. The contribution of pediatric surgeon and neurosurgeon to the specific operations is shown in Table 2. Through this interdisciplinary approach a macroscopically complete resection confirmed by free tumor margins (R0) could be achieved in 22/25 cases.

**Table 2:** Surgical data

ID	Diagnosis	Contribution pediatric surgeon	Contribution neurosurgeon	Neuromonitoring (yes/no)	Resection status	Postoperative Complications
1	AL	Tumor resection	Dissection median nerve Dissection ulnar nerve	No	R0	None
2	DT	Tumor resection	Reconstruction ulnar nerve (sural nerve grafting)	Yes	R0	None
3	DT	Tumor resection	Exposure nerve roots S1-S2 Resection within sacral plexus Neurolysis femoral and obturator nerves	Yes	R0	None
4	ES	Tumor resection	Dissection of brachial plexus Dissection of nerve roots C8-T1	No	R0	Horner Syndrome Sensorial and motoric deficits C8-T1
5	GN	Tumor resection	Dissection sciatic nerve	Yes	MCR	None
6	IF	Tumor resection	Dissection brachial plexus Dissection axillar nerve Dissection phrenic nerve	Yes	R0	None
7	MPNST PNF PNF	Tumor resection	Resection alongside nerve root L5 Resection neurofibroma median nerve Resection neurofibroma peroneal nerve	Yes	R0	Transient foot flexor paresis
8	MPNST	Tumor resection	Resection of nerve root L3	Yes	R0	Unilateral paresis of iliopsoas
9	NB	Tumor resection	Dissection and resection tumor neuroforamina T11-T12	No	MCR	None
10	NB	Tumor resection	Dissection nerve root L3	Yes	MCR	None
11	NB	Tumor resection	Dissection sciatic nerve Dissection obturator nerve	Yes	MCR	None
12	NB	Tumor resection	Dissection right sacral nerves	Yes	MCR	None
13	NB	Tumor resection	Dissection femoral nerve Dissection obturator nerve Dissection lumbo-sacral plexus	Yes	MCR	None
14	PNF	Resection neurofibroma	Dissection brachial plexus	No	MCR	None
15	PNF	Resection neurofibroma (thoracoscopic)	Resection axillar tumor extension	No	MCR	None
16	PNF	Resection neurofibroma (thoracoscopic)	assistance to pediatric surgeon	No	MCR	None
17	PNF	Exposure neurofibroma	Resection neurofibroma	No	MCR	None
18	PNF	Exposure neurofibroma Mobilization urinary bladder	Resection neurofibroma	Yes	MCR	None
19	PNF	Exposure neurofibroma	Resection neurofibroma	No	MCR	None
20	PNF	Resection neurofibroma (thoracotomy)	assistance to pediatric surgeon	No	MCR	None
21	PNF	Exposure neurofibroma	Resection neurofibroma Dissection intervostal nerve XII	Yes	MCR	None
22	PNF	Exposure retroperitoneal structures	Resection neurofibroma	No	nMCR	None
23	PNF	Exposure retroperitoneal structures	Resection neurofibroma	No	MCR	None
24	PNET	Tumor resection	Partial excision soleus muscle Dissection sciatic nerve Dissection tibial nerve Dissection peroneal nerve	Yes	R0	None
25	RMA	Tumor resection	Dissection median nerve	No	R0	None

Resection status: *MCR* macroscopically complete resection, *nMCR* near macroscopic complete resection, *R0* complete resection (macroscopically and microscopically), *AL* angiolipoma, *DT* desmoid tumor, *ES* Ewing sarcoma, *GN* ganglioneuroma, *IF* infantile fibromatosis, *MPNST* malignant peripheral nerve sheath tumor, *NB* neuroblastoma, *PNF* neurofibromatosis type I associated plexiforme neurofibroma, *PNET* peripheral neuroectodermal tumor, *RMA* alveolar rhabdomyosarcoma

The remaining 3 patients underwent an incomplete primary resection (macroscopic residuals, R2). In one patient diagnosed with a NF1 associated neurofibroma of the retroperitoneal region, an intracapsular tumor removal was carried out since no capsular infiltration was encountered. However, the final histological result revealed a malignant peripheral nerve sheath tumor (MPNST). Fourteen days later, a second-look procedure was performed and the retroperitoneal tumor bed was completely resected together with the root of L3 nerve (no microscopic residuals, R0). Another patient with a suspected thoracic neuroblastoma at the level of C7-T1 underwent an emergency procedure mandated by the compression of the trachea. A subtotal tumor resection through a trap-door incision was achieved. The tumor parts which infiltrated the neuroforamina and the brachial plexus were left in place. The histological examination revealed Ewing sarcoma. An interdisciplinary pediatric surgical/neurosurgical second-look procedure was carried out 7 days later, and a complete tumor resection together with resection of the second rib, exposure of the nerve roots C8-T1 and brachial plexus as well as resection of tumor components involving the neural structures could be achieved. In the third patient with an extensive plexiforme neurofibroma of the retroperitoneal region, a complete tumor resection could not be achieved. Due to the benign nature of the remaining manifestations on imaging, an incomplete resection was accepted. The final histological examination confirmed benign neurofibromas and no further surgery was indicated.

**Fig. 3:**
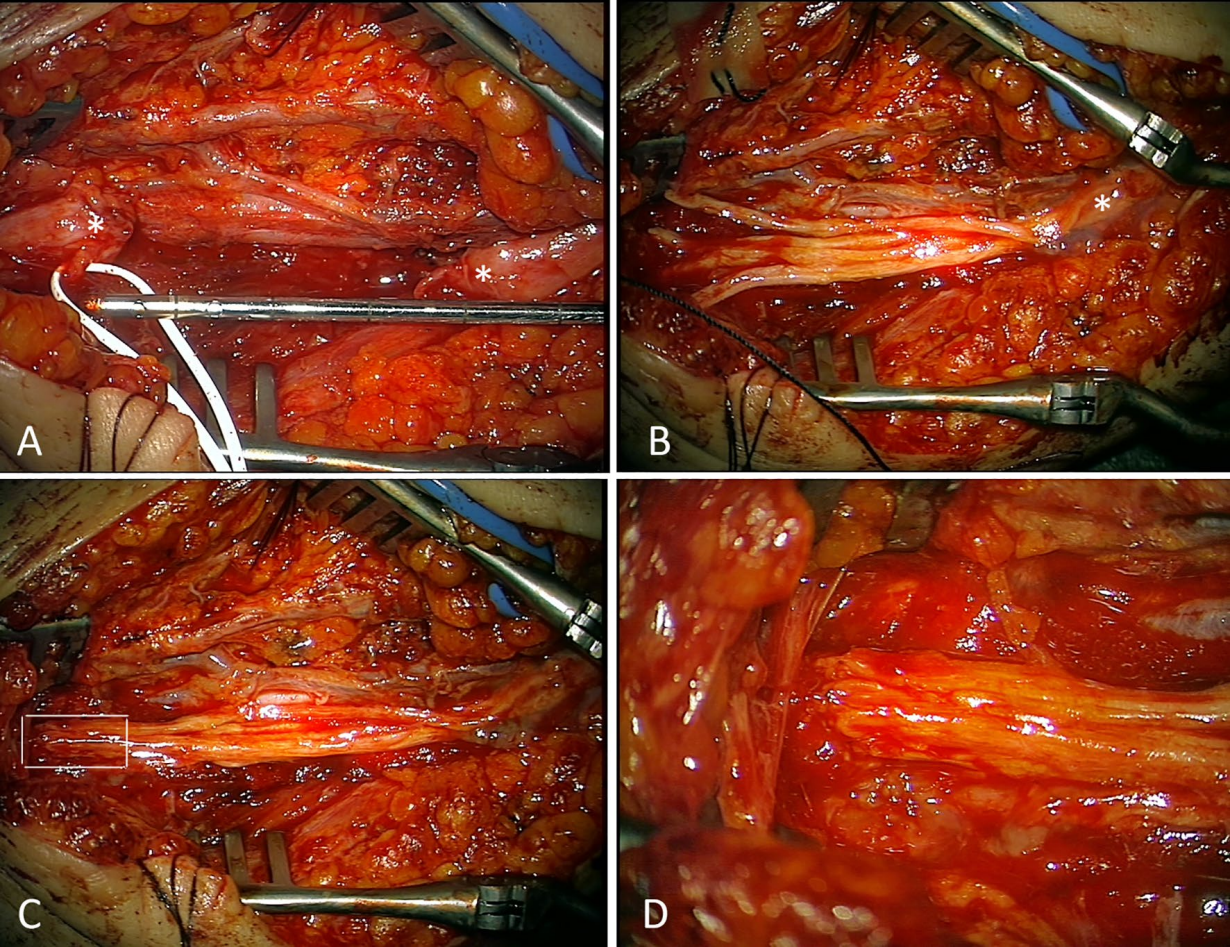
Reconstruction of ulnar nerve after tumor resection. (**A**) Defect between nerve endings (*) after tumor resection of 4 cm which increases after proper nerve end trimming to 5 cm; (**B**) 4 sural nerve grafts have been positioned and are already fixed to one nerve end by four 10.0 sutures and covered with fibrin glue for additional stability of the anastomosis (*); at the other end the nerve roods are still waiting for adaptation; (**C**) Final situation after microsurgical sutures for adaption prior to fibrin glue application. White rectangle depicts area of detail image; (**D**) Details of microsurgical anastomosis of 4 transplants to nerve stump. 10.0 sutures are barely visible as little black spots

**Fig. 4:**
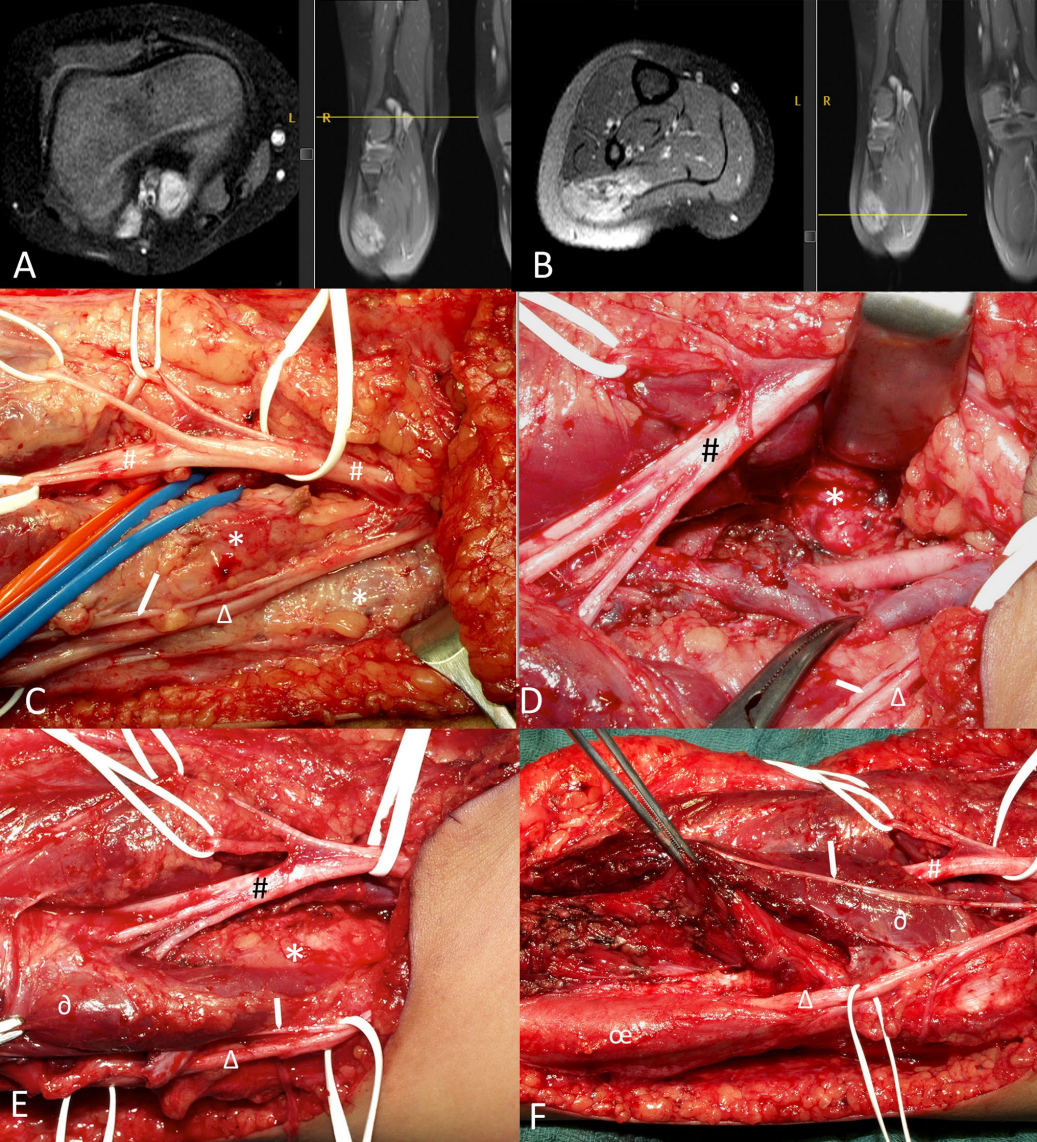
Intraoperative image of a patient with PNET of the right popliteal and upper calf showing the complex exposure of tumor inbetween the right tibial nerve and it’s branches,, the sural nerve, the peroneal nerves and the popliteal vessels. (**A**) Upper part of tumor (*) in the poplitea corresponding to intra-operative images (**C**–**E**), located in between nerves and vessels, laterally and medially of the vascular bundle; (**B**) Lower part of the tumor infiltrating the lateral gastrognemius muscle but sparing the peroneal muscles, corresponding to F; (**C**) Exposure of the poplitea. *tumor; ^**#**^tibial nerve (thick white rubber band) and superficial skin branches (thin white rubber bands); ***∆***: peroneal nerve; → : sural nerve. Blue and red rubber band lead to popliteal vein and artery; (**D**) Situation after resection of large tumor component in between nerves and vessels. The tibial nerve (**#**) and its three motor branches to the lateral and medial gastrognemuis heads and the soleus muscle is elevated with the retractor to visualis the tumor part (*) dorso-medially of the exposed popliteal artery and vein in the middle of the image. **∆**: peroneal nerve; → : sural nerve; (**E**) Upper calf region where the tibial (#) nerve branches enter the gastognemius and soleus muscles. *: tumor; **∂**: upper lateral head of gastrognemius muscle; **∆**: peroneal nerve; → : sural nerve; (**F**) At the end of surgery after resection of the tumor within the lateral head of the gastrocnemius muscle. The upper part of the muscle (**∂**) could be preserved. The peroneus muscle (**œ**), the peroneal nerve (**∆**) and sural nerve (** →**) remain unaffected. ^**#**^tibial nerve

In two patients with tumors of the limbs an amputation was planed at outside institutions. These children were referred to our institution after central surgical review. The interdisciplinary pediatric surgical/neurosurgical approach enabled a limb-sparing complete tumor resection in both patients. In one patient with desmoid tumor of the left elbow a reconstruction of the infiltrated ulnar nerve with two 25 cm sural nerve grafts was performed after en-bloc tumor resection (Fig. 3). The other patient had a PNET tumor of the right lower leg. After a complex exposure and microsurgical separation (using high-magnification microscope) including frequent use of bipolar peripheral nerve stimulator at low currents of the right sciatic nerve division, right tibial nerve, as well as the superficial and profound peroneal nerves from the tumor, a complete resection of the mass could be achieved (Fig. 4).

### Neurological outcome

In 17/25 patients, no preoperative functional nerve deficit was present and no deficits occurred postoperatively despite nerve attachment/encasement by the tumor.

Unexpected postoperative neurological deficits occurred in 2/25 patients (grade I, Dindo-Clavien classification(Dindo et al. 2004). One patient developed a transient peroneal paresis after resection of a neurofibroma of the peroneal nerve. A complete restoration of the function was observed during follow-up. Another patient developed a persistent Horner syndrome and a transient senso-motor deficit of C8-T1 after resection of an intrathoracic plexiforme neurofibroma.

The patient with retroperitoneal MPNST, by whom the nerve root L3 had to be sacrificed during second-look surgery, had consequently an expected and calculated persistent paresis of the right iliopsoas muscle. However, the functional deficits improved by intensive physiotherapy.

In 6/25 patients, a preoperative functional deficit was present. In 2 of these 6 patients, the deficit partially improved after the operation. However, none suffered additional nerve function deficits. Details of these 6 cases are as follows:

In the patient with desmoid tumor of the left forearm, who already had complete ulnar and radial nerve deficits, the ulnar sensory deficits improved after ulnar nerve reconstruction. However, despite return of some voluntary muscle action potential in abductor digiti minimi muscle, no functional improvement of ulnar nerve motor hand function was seen due to long-standing ulnar paresis and severe preoperative muscular atrophy.

The second patient had an angiolipoma of the right elbow. The tumor infiltration and chronic inflammation of the articular capsule and the biceps tendon led to a preoperative fixation of the right elbow in a 90° position. The range for flexion and extension was at most 20°. Three months after the complete removal of the tumor and intense physiotherapy, the range of motion improved significantly (almost 70°).

In one patient with ganglioneuroma of the pelvis involving the nerve roots L5-S3, voiding dysfunction already existed preoperatively. Postoperatively, no improvement of bladder function was observed, but also no new senso-motor deficits in the legs. Clean intermittent catheterization was initiated.

One patient with neuroblastoma of the pelvis with penetration into the sacral hiatus through the foramina S2-S4 and voiding dysfunction required a permanent suprapubic urinary catheter because of persisting voiding dysfunction after surgery. No deficits to sensori-motor S2 function occurred.

In another patient with pelvic neuroblastoma and involvement of the right L5-S4 nerves an increased external rotation of the right leg, reduced dorsal flexion of the right foot, and pes equinus of the right foot existed preoperatively. Postoperatively no improvement but also no worsening or new deficits occurred.

One patient with desmoid tumor of the left pelvis, who had been operated at an outside institution before being referred to us, presented with an impaired dorsal flexion of the foot because of a lesion of the peroneal part of the sciatic nerve, respectively the root of L5. Following surgery at our institution no improvement but also no worsening of the neurological status or new deficits occurred.

### Tumor relapse

Local tumor relapse occurred in 4 patients. In one patient with neuroblastoma of the pelvis a local relapse occurred 29 months after operation. This was managed by radiotherapy, however, this patient died 38 months after the initial operation.

The patient with desmoid tumor of the left forearm and elbow region developed three local relapses at 5, 13, and 17 months after surgery. The relapses were distant to the reconstructed nerves and vasculature and were treated by surgery (relapse 1 and 2) and radiotherapy (relapse 3) without causing new neuronal deficits.

In the patient with a desmoid tumor of the pelvis, a local relapse occurred 12 months postoperatively and was treated by surgery (no new deficits were encountered).

The patient with a PNET tumor of the right lower leg developed a local relapse 14 months after surgery, distant to neurovascular structures, and was treated by resection and postoperative radiotherapy.

## Discussion

Surgery plays a key role in local control of pediatric solid tumors (Cecchetto et al. 2005). In neuroblastoma, the most prevalent intraabdominal tumor in the pediatric population, the completeness of resection has been shown to correlate with improved overall survival (Rich et al. 2011; La Quaglia et al. 2004; Fischer et al. 2017). MPNST tumors in NF1 patients are virtually insensitive to radiotherapy and to chemotherapy and they can only be cured by radical resection (Reinert et al. 2019). In patients with Ewing sarcoma, microscopically complete tumor resection should be achieved in all patients (Seitz et al. 2019). In patients suffering from desmoid tumors, in the case of subsequent progression or a significant increase in the symptom burden, surgical resection may be the initial therapy approach and should aim a wide microscopic margin resection (Kasper et al. 2020). When positive microscopic margins are anticipated, then treatment options other than surgery may be preferred (Kasper et al. 2020). Even though the risk of local recurrence seems to be lower after a combined treatment, the outcome after surgery combined with radiotherapy prove not to have statistically significant difference compared to surgery alone. Additionally, the morbidity of the two combined modalities is higher (Kasper et al. 2020; Gluck et al. 2011). Therefore, complete tumor resection remains the main objective for the surgical treatment of children suffering from solid tumors and represent a prognostic factor for survival in many entities. Given that tumor resection may reduce potential late effects of chemotherapy or radiotherapy, surgeons are challenged to do their best (Cecchetto et al. 2005).

Safety and completeness of resection depends on multiple factors, of which the most important are tumor size and location, relationship to adjacent vital structures (vessels, nerves, and organs) as well as the experience and versatility of the surgeons (Cecchetto et al. 2005). The strategy for complete tumor resection may be straight forward if the tumor is limited to an organ such as the kidney, liver, adrenal gland or ovary. However, tumor entities such as neuroblastomas, rhabdomyosarcomas, tumors of the Ewing sarcoma family, but also desmoid tumors, have a great propensity to encase or infiltrate important vascular, as well as neural structures. In these tumors, the resection might become surgically challenging. While a neuroblastoma may be resected in fragments after dissection and exposure of vascular structures, in patients with soft tissue sarcomas rupture of tumor capsule should be avoided by all means and an en-bloc resection is required. This task may be accomplished without difficulty if the tumor is limited to a certain compartment. If essential structures (vessels and nerves) are affected in form of infiltration or encasement, the probability of an incomplete resection is high and surgery may be complicated by injury of these vital structures (Warmann et al. 2011, 2012). Surgical resection of these tumors regularly necessitates vascular reconstruction, separation of nerves from tumor tissue including perineurectomy, and in certain circumstances of true nerve infiltration, resection and reconstruction. Reconstruction of resected nerves can avoid mutilating operations. If not treated in centers with high expertise in interdisciplinary pediatric surgical oncology, some of these children may be subjected to either mutilating procedures or—even worse—may be regarded as inoperable. Reports that systematically analyze and describe the rate of mutilating surgical procedures in children are rare. Antoniello et al. reported on a rate of 12% of mutilating surgical procedures in children suffering from non-chemo-sensitive pediatric soft tissue sarcoma treated within the Italian Cooperative Group Studies RMS-79 (Antoniello et al. 2003). In our study cohort, limb amputation could be avoided in two patients.

Preoperative imaging plays a key role in planning of the surgical strategy. Special additional information can be derived from PET/MR or PET/CT. PET/MR imaging offers the diagnostic advantage of multiparametric characterization of pathophysiologic processes combined with a significant reduction in radiation exposure of about 50–75% compared to CT or PET/CT imaging (Gatidis et al. 2017, 2016; Schäfer et al. 2020). However, reading and interpreting PET/MR data are a complex task, as numerous sequences have to be analyzed together with PET data. This requires a high level of expertise in pediatric MRI and pediatric nuclear medicine. In our institution, examinations are interpreted in consensus by multidisciplinary teams consisting of a radiologist and a nuclear medicine specialist and the results are presented and discussed at the institutional interdisciplinary pediatric tumor board (Gatidis et al. 2016).

However, with regard to nerve involvement, standard imaging workup with MRI is often not conclusive enough and sometimes a clear distinction between infiltration, encasement or displacement of affected nerves cannot be made.

Nerve ultrasound is an emerging tool in neuromuscular disorders, particularly in nerve trauma, entrapment syndromes, and polyneuropathies as it allows visualization and localization of morphological changes (Schubert et al. 2020). Nerve pathologies detectable by ultrasound include enlargement or diminution of cross-sectional areas of nerves, loss of fascicular architecture, increase or decrease of nerve echo-structure and change of perineural tissue as well as mobility of nerve against surrounding structures (Schubert et al. 2020). The potential of nerve ultrasound in the diagnostic and preoperative workup of nerve tumors has already been shown for NF associated tumors (Winter et al. 2020, 2017).

In our institutional experience, the superior visualization potential of high-resolution nerve ultrasound is able to depict all surgically relevant details also in tumors arising from outside the nerve and thus has a profound influence on our surgical planning and preoperative counseling of parents and patients. Due to the limited depth penetration of high-resolution ultrasound the methodology is not applicable to intrathoracic, intra- and retroperitoneal or pelvic pathologies. For specific tumor locations, we strongly recommend preoperative interdisciplinary investigations with nerve sonographer and surgeon side-by-side.

It is of paramount importance that the decision toward extended or even extreme surgical procedures has to be made on an interdisciplinary basis and has to balance the aimed surgical success against a functional or oncological outcome, additionally impaired by the occurrence of surgical complications during or after operation (Warmann et al. 2012). Indications should be made in the context of an integrated treatment strategy established by a multidisciplinary tumor board (MDT) which should include the pediatric oncologist, pediatric radiologist, pediatric surgeon, nerve sonographer, radio-oncologist, pathologist, and (pediatric) neurosurgeon or plastic surgeon with expertise in peripheral nerve surgery. Whereas surgical strategies are usually standardized in standard-risk tumors, they are mostly individual and depending on several momentary aspects in advanced tumor diseases. Regularly, developing a surgical strategy for complex tumors is highly complicated and requires extensive planning (Warmann et al. 2012).

Using the described interdisciplinary pediatric surgical-neurosurgical approach a complete tumor resection was finally achieved in 24/25 patients with very good functional outcome and given the complexity of cases a very low rate of associated complications.

This study has several limitations. One limitation is in the small number of patients and the heterogeneity of the study population. However, taking into account the rareness of the different malignancies, the cohort described in this study is of clinical and scientific relevance. Another limitation resides in the paucity of the literature addressing this topic. To our knowledge, this is the first study in the literature addressing an interdisciplinary surgical approach in specific conditions of pediatric solid malignancies.

In conclusion, interdisciplinary pediatric surgical/neurosurgical operations performed by experienced surgeons in centers of excellence for treatment of children with complex solid tumors achieve a very high rate of complete tumor resection with good surgical, functional, and oncologic outcomes. This example might serve as model for other conditions as well for example in cases of complex vascular involvement.
